# Supplementary material for: Empirical methods for controlling false positives and estimating confidence in ChIP-Seq peaks
Source: BMC Bioinformatics. 2008 Dec 5;9:523. doi: 10.1186/1471-2105-9-523 (PMC2628906; doi:10.1186/1471-2105-9-523)
Supplement: Additional File 1 — A variety of html documents from the USeq web site detailing the available applications, their best usage, output file type descriptions, command line menus, etc. [file 1471-2105-9-523-S1.zip › USeqUserGuides/JavaDoc.zip!/JavaDoc/edu/utah/seq/data/package-use.html]

Uses of Package edu.utah.seq.data


|  |  |  |
| --- | --- | --- |
| **Overview** \| **Package** \| Class \| **Use** \| **Tree** \| **Deprecated** \| **Index** \| **Help** | |  |
| PREV   NEXT | **FRAMES**    **NO FRAMES**     **All Classes** |  |


---


## **Uses of Package edu.utah.seq.data**

| Packages that use edu.utah.seq.data | |
| --- | --- |
| **edu.utah.seq.analysis** | Primary analysis applications and helper classes for working with binary PointData, xxx.bar.zip files. |
| **edu.utah.seq.data** | Helper classes for working with binary PointData, EnrichedRegions, etc. |
| **edu.utah.seq.parsers** | Parsers for converting sequencing data into binary PointData, xxx.bar.zip files. |

Classes in edu.utah.seq.data used by edu.utah.seq.analysis

****EnrichedRegion****

****Info****
  
          Data file information.

****SmoothingWindow****
  
          Coordinates are interbase!

****SmoothingWindowInfo****
  
          Chromosome specific container for window level results from EnrichedRegionMaker.

Classes in edu.utah.seq.data used by edu.utah.seq.data

****Info****
  
          Data file information.

****Point****

****PointData****
  
          Container for holding genomic positions - scores data.

****SmoothingWindow****
  
          Coordinates are interbase!

****SmoothingWindowInfo****
  
          Chromosome specific container for window level results from EnrichedRegionMaker.

Classes in edu.utah.seq.data used by edu.utah.seq.parsers

****Point****

****PointData****
  
          Container for holding genomic positions - scores data.

---


|  |  |  |
| --- | --- | --- |
| **Overview** \| **Package** \| Class \| **Use** \| **Tree** \| **Deprecated** \| **Index** \| **Help** | |  |
| PREV   NEXT | **FRAMES**    **NO FRAMES**     **All Classes** |  |


---
